# A Scanning Transmission X-ray Microscopy Study of Cubic and Orthorhombic C_3_A and Their Hydration Products in the Presence of Gypsum

**DOI:** 10.3390/ma9090745

**Published:** 2016-08-31

**Authors:** Vanessa Rheinheimer, Sejung Rosie Chae, Erich D. Rodríguez, Guoqing Geng, Ana Paula Kirchheim, Paulo J. M. Monteiro

**Affiliations:** 1 Berkeley Education Alliance for Research in Singapore–BEARS, Singapore 138602, Singapore; 2 Department of Civil and Environmental Engineering, University of California, Berkeley, CA 94720, USA; busyrosy@berkeley.edu (S.R.C.); guoqing_geng@berkeley.edu (G.G.); monteiro@berkeley.edu (P.J.M.M.); 3 Department of Civil Engineering, Federal University of Rio Grande do Sul, Porto Alegre 90035-190, RS, Brazil; anapaula.k@ufrgs.br (A.P.K.); erichdavidrodriguez@gmail.com (E.D.R.); 4 Polytechnic School of Civil Engineering, IMED, Passo Fundo 99070-220, Brazil

**Keywords:** tricalcium aluminate, gypsum, scanning transmission X-ray microscopy, hydration

## Abstract

This paper shows the microstructural differences and phase characterization of pure phases and hydrated products of the cubic and orthorhombic (Na-doped) polymorphs of tricalcium aluminate (C_3_A), which are commonly found in traditional Portland cements. Pure, anhydrous samples were characterized using scanning transmission X-ray microscopy (STXM), X-ray photoelectron spectroscopy (XPS) and X-ray diffraction (XRD) and demonstrated differences in the chemical and mineralogical composition as well as the morphology on a micro/nano-scale. C_3_A/gypsum blends with mass ratios of 0.2 and 1.9 were hydrated using a water/C_3_A ratio of 1.2, and the products obtained after three days were assessed using STXM. The hydration process and subsequent formation of calcium sulfate in the C_3_A/gypsum systems were identified through the changes in the L_III_ edge fine structure for Calcium. The results also show greater Ca L_II_ binding energies between hydrated samples with different gypsum contents. Conversely, the hydrated samples from the cubic and orthorhombic C_3_A at the same amount of gypsum exhibited strong morphological differences but similar chemical environments.

## 1. Introduction

The most important crystalline phases of clinker Portland are tricalcium silicate (3CaO·SiO_2_ referred in the chemistry of cement as C_3_S), dicalcium silicate (2CaO·SiO_2_ or C_2_S), tricalcium aluminate (3CaO·Al_2_O_3_ or C_3_A) and ferrite (4CaO·Al_2_O_3_·Fe_2_O_3_ or C_4_AF). Although the tricalcium aluminate content in clinker Portland is relatively low (~5–10 wt %), its behavior is crucial as it controls the setting time during cement hydration [1]. The presence of minor elements, mainly Na^+^, K^+^, Mg^2+^, and Fe^3+^, during the clinkering modifies the structure of C_3_A, and the alkali content (mainly Na^+^) derive from the raw materials and/or fuels during the clinkering are relatively low, resulting in two C_3_A polymorphs [2]. Pure C_3_A exists in cubic form with a lattice constant of 15.263 Å and space group Pa3, containing 264 atoms built up from hollow rings of six corner sharing AlO_4_ tetrahedra that are held together by Ca^2+^ ions [3]. The behavior of the C_3_A solid solution with Na_2_O has been reported widely where the content of Na^+^ plays an important role in the lattice constant due to the Ca^2+^ substitution. The phase obtained exhibits the general formula Na_2*x*_Ca_3-*x*_Al_2_O_6_ [4]. This cubic structure is stable up to the Na_2_O content of 2.4%. Conversely, the C_3_A synthesis containing Na_2_O between 4.6 wt % and 5.7 wt % leads to an orthorhombic structure (which is also referred here as Na-doped C_3_A). A greater content of Na_2_O (>5.7 wt %) changes the crystal structure to monoclinic [5]. In ordinary Portland cements (OPC), the C_3_A appears in its cubic structure or in combination with an orthorhombic structure. Monoclinic C_3_A is not identified due to the low content of alkalis during clinkering. Therefore, if sufficient alkalis are available, the amount of orthorhombic C_3_A that is formed will be greater and is enhanced by rapid clinker cooling [1].

The reaction of C_3_A with water is almost instantaneous, releasing large amounts of heat (1260 J/g for the pure phase) and hindering the possibilities of use in situ when mixing, transporting, and placing the concrete [6,7]. The hydrated products formed are calcium aluminate hydrates (Ca_2_[Al(OH)_5_]_2_·3H_2_O or C_2_AH_8_, 2[CaAl(OH)_7_·3H_2_O or C_4_AH_13_ and 4CaO·Al_2_O_3_·19H_2_O or C_4_AH_19_), which are metastable phases that are later transformed into a stable hydrogarnet product (3CaO·Al_2_O_3_·6H_2_O or C_3_AH_6_) [8,9]. The flash set of C_3_A is controlled by the addition of calcium sulfate (~5 wt % with respect to the overall content of the cement) to slow down the reaction of C_3_A with water and to improve the concrete workability. The presence of sulfates strongly affects the hydration mechanism of the C_3_A, and two stages of reaction can be identified: initially the reaction between the C_3_A, sulfates and water forms hydrous calcium alumina-sulfate, or ettringite (Ca_6_Al_2_(SO_4_)_3_(OH)_12_·26H_2_O, AFt). As the calcium sulfate content decreases, the remaining C_3_A reacts with water, and the previously formed ettringite forms calcium monosulfoaluminate hydrate (Ca_4_Al_2_SO_4_(OH)_12_·6H_2_O, AFm) and hemicarboaluminate when exposed to CO_2_.

Previous reports have shown that the hydration between cubic C_3_A and orthorhombic C_3_A occurs in different manners, forming different hydration products with distinct morphologies [10,11,12,13,14,15]. Orthorhombic C_3_A shows the highest reactivity in the absence of gypsum and high susceptibility to sorb water even at low relative humidity (<55%) [16]. ^27^Al-nuclear magnetic resonance (NMR) analysis shows significant differences in the Al dissolution between the hydrated products of cubic and orthorhombic C_3_A within a saturated solution of calcium hydroxide and gypsum. In this sense, real time hydration studies with high resolution X-ray microscopy also identified that orthorhombic C_3_A reacts faster than cubic C_3_A and the corresponding formation of ettringite as a main hydrated product. Likewise, the ettringite crystals formed from the hydration of orthorhombic C_3_A are larger and have a lower aspect ratio when compared to the products obtained from the hydration of cubic C_3_A [10]. Based on isothermal calorimetric results, the dissolution process of C_3_A and the initial formation of AFt can be retarded at early ages of reaction (<2 h) as the content of Na_2_O within the structure is greater [11]. However, the inclusion of greater amounts of sulfates promotes the formation of ettringite crystal (needle like hydrates). At very early stages, the hydration of orthorhombic C_3_A forms a gel-type product surrounding the particles, which appears to be transformed later into a needle-like ettringite phase [10].

Understanding C_3_A reactivity during the hydration of Portland cement is essential for improving concrete workability and for solving issues related to setting times and the development of organic admixtures. Taking into account the different behavior identified for the distinctive C_3_A polymorphs in the presence or absence of gypsum, the cubic/orthorhombic C_3_A ratio present in PC will have a strong effect on the kinetic of the reaction and the subsequent time of setting. Differences in the effectiveness of different organic admixtures (mainly plasticizer and superplasticizers) in the cubic and orthorhombic C_3_A were observed [12,17,18], and a better understanding about their hydration mechanisms will contribute to the development of more suitable and higher performance products. Therefore, this paper shows a detailed assessment of their hydration products in a calcium sulfate solution at different ratios using scanning transmission X-ray microscopy (STXM). STXM combines spectroscopy and microscopy by imaging with spectral sensitivity and concurrently recording spectra from very small spots with a spatial resolution on the nanometer scale. A monochromated X-ray beam produced by synchrotron radiation is used and provides quantitative information on the specimen’s local elemental and chemical composition and magnetization. In addition to obtaining the chemical speciation information, STXM allows identifying heterogeneity within the samples on a particle-by-particle basis. The use of STXM along with near-edge X-ray absorption fine structure (NEXAFS) spectra allows the chemical characterization and identifying spatial heterogeneities, providing a specific element quantification of individual species with a high spectral resolution [19]. This technique is well suited for nano-structured or amorphous materials or compounds, such as the main hydrated phases in OPC (C–S–H) as it allows assessing the oxidation states, bond length, coordination numbers and neighboring atoms of the X-ray absorber atom of interest. Therefore, STXM has become an important tool for the assessment of the immobilization process in cementitious systems as well as the development of microstructural modeling, and its suitability for this type of material is described in the literature [20,21,22,23,24,25,26]. This paper assesses Ca speciation in anhydrous cubic C_3_A and orthorhombic C_3_A and their corresponding hydrated products in presence of gypsum using STXM coupled with NEXAFS spectroscopy. Furthermore, unhydrated pure C_3_A phases were assessed using X-ray photoelectron spectroscopy (XPS) to validate the chemical state of the elements present in their structures, and X-ray diffraction (XRD) is used for phase identification.

## 2. Experimental and Methods

### 2.1. Materials

Cubic (pure) and orthorhombic (Na-doped) C_3_A were supplied by CTL, Inc., Skokie, IL, USA. Purity is not provided but XRD assures the phases observed. Gypsum (CaSO_4_·2H_2_O) used in this study was provided by Fisher Scientific (Waltham, MA, USA) with 98% purity.

### 2.2. Experimental

Particle size distributions of the samples were measured in a CILAS 1180 Laser Granulometer (Cilas, Orléans, France) in 99.5% pure liquid isopropyl alcohol. The lower size limit of this equipment was 40 nm, and the Fraunhofer approximation was applied in the analysis of the granulometry data.

Mineralogy was analyzed by X-ray diffraction using a PANalytical Empyrean diffractometer with Cu Kα (λ = 1.5418 Å) radiation, step size 0.017° with ~20 s count per step. X-ray diffraction (XRD) (PANalytical XPert Pro, Cu Kα radiation with step size of 0.02° 2θ, PANalytical, Almelo, The Netherlands). Quantitative analysis was performed through Rietveld analysis using the X’Pert High Score Plus software (PANalytical, Almelo, The Netherlands), with goodness-of-fit calculated to achieve a value higher than 9 and Weighted R profile maximum 8%. The procedures suggested by Gobbo [27] and Post and Bish [28] were adopted to perform the refinement. Inorganic Crystal Structure Database (ICSD) for cubic C_3_A, hydrogarnet, orthorhombic C_3_A, and monohydrocalcite were used for refinement and quantification of the anhydrous crystalline phases. The background was fitted with a Chebyshev function with 4 terms. Lattice constants, phase fraction, and zero shift were the parameters refined. The software used a Pseudo-Voigt algorithm for minimizing the residue Ry (*x*) and modeling the peaks.

SPECS™ X-ray photoelectron spectroscopy (XR50 Al K∝ X-ray source operated at 150 W and a Phoibos MCD-9 detector (SPECS, Berlin, Germany)) were used to confirm the mineralogical composition, purity and condition of the non-hydrated C_3_A. For the XPS, the spectra were recorded with a pass energy of 25 eV using a 0.1 eV step size at a pressure below 10^−9^ mbar. The powder material was pressed into pellets, and samples were fixed onto holders with a copper tape. General scans were repeated three times. Additionally, specific high-resolution scans were carried out for elements of interest including calcium, aluminum, carbon, oxygen, and sodium, which were previously identified in the general scans. Data were extracted from the spectra via peak fitting using Casa XPS™ software. A Shirley background and the Gaussian–Lorentzian 30 line shape were assumed in all cases.

The STXM characterization of both the cubic and orthorhombic C_3_A was carried out using two different conditions: (1) pure unhydrated C_3_A samples (cubic and orthorhombic); and (2) C_3_A/gypsum blends with a mass ratio of 1:0.2 and 1:1.9. Pure gypsum was analyzed for comparison. The water content was adjusted to obtain a water/C_3_A ratio of 1.2, and the mixture was then cured for three days in a sealed plastic container with a relative humidity of ~90% at ~25 °C. The hardened samples were ground in an agate mortar and diluted in ethanol. A micro volume of the suspension was dropcast on a 0.5 × 0.5 mm and 100-nm-thick silicon nitride window embedded in the center of a 3 × 3 mm silicon frame. The STXM analysis was carried out at beamline 5.3.2.2 of the Advanced Light Source (ALS) at the Lawrence Berkeley National Laboratory. Specimens were loaded in the experimental chamber and quickly vacuumed (up to 10^−2^ Torr). Helium was then introduced for protection purpose at 250 Torr. Line scans and image stacks were run on the areas of interest with an output energy-dependent absorption signal with a 1D and 2D spatial resolution, respectively. The energy scanning range was set to 340–360 eV with a dwell time between 0.6 and 0.8 ms. The results were analyzed using the software aXis 2000 (McMaster University, Hamilton, ON, Canada) [29], and the normalization and background subtraction for the spectra were performed by dividing each sample’s spectrum by the spectrum of an area with no particles (I_0_). The elemental maps were obtained by subtracting the absorption images below the absorption edge from that above the edge, which also allows for alignment of image stacks and extraction of the NEXAFS spectra from the image stack and the line scan measurements. A spectra peak deconvolution provided the exact absorption fine structure for each peak, which could be observed by a local maximum, using Gaussian and Lorentz line shapes. Taking into account that the sample thickness can be a restriction in the assessment, where in thicker and denser samples, the material absorbs all the radiation and not enough transmission is detected; therefore, the smallest particles and highly monochromated light were selected for the STMX analysis [30].

## 3. Results and Discussion

### 3.1. Characterization of Pure Phases

#### 3.1.1. Particle Size Distribution

The results showed a particle size distribution with d (0.90) < 30 μm and a mean particle size of 11.0 μm and 14.0 μm for C_3_A-orthorhombic and C_3_A-cubic, respectively. Gypsum presented a particle size distribution with d (0.90) < 39 μm and a mean particle size of 19.0 μm.

#### 3.1.2. X-ray Diffraction

**Figure 1:**
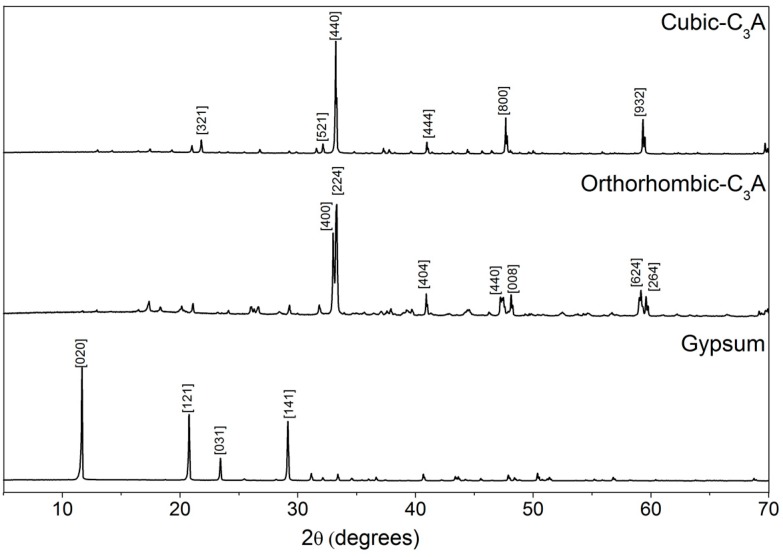
Cu Kα diffractogram of the anhydrous cubic C_3_A, the orthorhombic C_3_A and the gypsum.

The XRD of the cubic C_3_A shows a cubic structure with a d-spacing at 2.69874 Å along the {440} directions (Figure 1). Conversely, the orthorhombic C_3_A exhibits the expected double refraction line at 2.692 Å and 2.714 Å along the {224} and {400} directions, respectively, which is characteristic of its orthorhombic structure. Comparisons of the XRD peak positions were made using data from Regourd et al. [31] (ICDD PDF No. 38-1429 for the cubic sample and ICDD PDF No. 26-0958 for the orthorhombic). The diffraction pattern for the gypsum is also shown in Figure 1.

X-ray diffraction (XRD) and Rietveld analyses showed that the C_3_A powder contained ≥99 wt % cubic C_3_A (ICSD# 1841, [32]) and ≥1 wt % of hydrogarnet (ICSD# 202316, [33]) (estimated uncertainty = ±5 wt %), ≥92 wt % orthorhombic C_3_A (ICSD# 1880, [34]), ≥7 wt % of hydrogarnet (ICSD# 202316) and ≥1 wt % of monohydrocalcite (ICSD# 100846, [35]) (estimated uncertainty = ±9 wt %).

#### 3.1.3. X-ray Photoelectron Spectroscopy (XPS)

The details of the spectral lines recorded using XPS are described in Table 1. The spectra were corrected for charging effects using the adventitious carbon peak at 284.8 eV.

**Figure 2:**
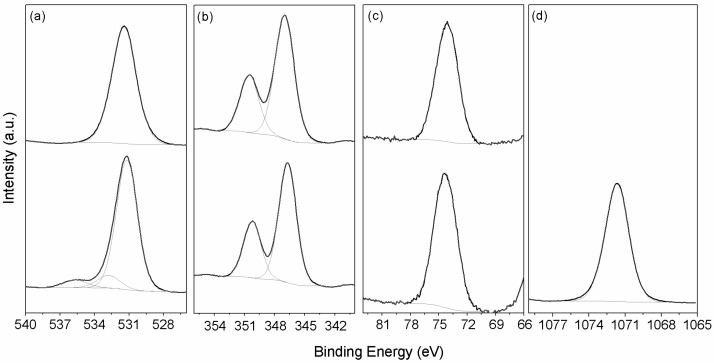
XPS spectra of the C_3_A cubic (**top**) and orthorhombic (**bottom**): (**a**) O 1s; (**b**) Ca 2p; (**c**) Al 2p; and (**d**) Na 1s.

A more detailed analysis of the surface region using XPS in the anhydrous cubic and orthorhombic C_3_A show slight differences in the chemical environments for the O, Ca and Al, which was obtained after the peak deconvolution of their corresponding XPS spectra (Figure 2, Table 2). The Al 2p peaks identified at the binding energy of 73.05 eV are assigned to the aluminate (Al species in tetrahedral coordination) [36,37]. Orthorhombic C_3_A shows a lower Ca 2p binding energy and a greater Al 2p binding energy than the cubic C_3_A (Table 2). This can be attributed to the fact that some Ca^2+^ ions are substituted by Na^+^, which also lead to three different peaks between 531.2 and 535.7 eV for the O 1s XPS spectra in the orthorhombic C_3_A sample, indicating the coexistence of different oxygen chemical species. The peak at higher energies is from oxygen bonded to the Na, whereas the peak at around 531.2 eV is likely related to the bonding with aluminum, also observed in the cubic C_3_A, as well as the peak at lower binding energy, which is likely to be a different chemical bonding with aluminum. It is observed that O 1s in cubic C_3_A presents lower binding energies than in orthorhombic C_3_A, following observations from [37]. The peak at 1071.7 eV for the Na 1s in the orthorhombic C_3_A also corroborates the presence of this element within the C_3_A phase. The C 1s signal detected at ~298.5 eV corresponds to the carbonate species due to the weathering of the samples.

The values of the binding energy for Al 2p and Ca 2p_3/2_ are coherent with other reports [38,39,40,41]. These results are aligned with the results presented by Dubina et al. [38,42] where the orthorhombic C_3_A showed a greater binding energy for Al 2p and lower values for Ca 2p_3/2_. However, in previous reports [43], unhydrated reference orthorhombic C_3_A shows a binding energy for Al 2p at 73.0 eV with partial hydration due to the environmental moisture increase the binding energy to 74.3 eV. However, it is necessary to take into account that the XPS probes only the surface of the sample (10 nm depth), which can be C_3_AH_6_. This behavior was also identified in the cubic C_3_A where a peak at 73.8 eV was observed after exposure to water vapor. These values are more in accordance to what is observed here. The Al 2p shift to higher binding energies is related to the formation of calcium aluminum hydrates (C–A–H), which indicates the possibility that the samples suffered a pre-hydration process prior to the analysis and during storage (even at lower humidity, ~55%). This result can be attributed to its greater susceptibility to partial hydration and the formation of hydrated phases on the surface and is proved by the thermogravimetric analysis results that show a loss of mass less than 8% and 1% for orthorhombic and cubic C_3_A, respectively. At the same time, the existence of a C 1s peak, which is in addition to the adventitious carbon peak, indicates the formation of calcium carboaluminates or calcium carbonates form free CaO. The higher intensity of C 1s peaks for the orthorhombic C_3_A elucidates a greater reactivity degree with CO_2_ during handling and storage [16], whereas Dubina et al. [37] describe, for their samples, also applicable here, that the surface of orthorhombic C_3_A can host the formation of large amounts of Na_2_CO_3_ from the carbonation of NaOH, being much more evident in these samples than in cubic C_3_A. This greater susceptibility to carbonation compared with cubic C_3_A leads to the formation of calcium monocarboaluminate and sodium carbonate. The greater reactivity and extended partial/superficial hydration for orthorhombic C_3_A is due to the presence of different energy δCa-Al separation and electronegativity caused by the inclusion of Na into the C_3_A lattice. Standard hydrated phases of C_4_AH_13_ and C_3_AH_6_ show Al 2p peaks at 73.8 eV and 74.3 eV, respectively [40,43], which can explain the pre-hydration signs by the higher binding energy of the samples in this study.

#### 3.1.4. Scanning Transmission X-ray Microscopy

**Figure 3:**
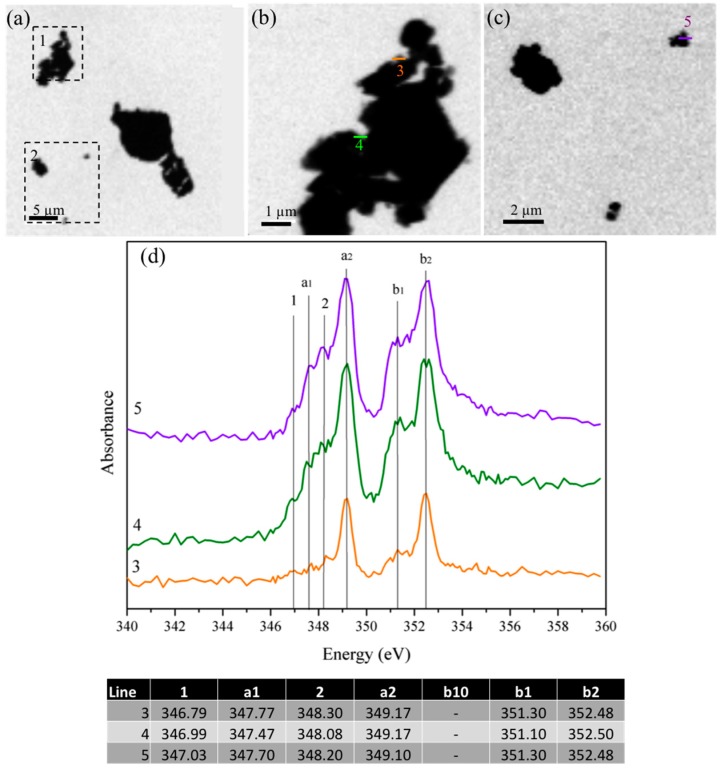
Image taken at the calcium L_II,III_ edge of the cubic C_3_A sample: (**a**) deneral overview of the particles; (**b**) detail of area 1 underlined in (**a**); (**c**) detail of area 2 underlined in (**a**); and (**d**) NEXAFS spectra at the Ca L_II,III_ edge for line scans 3–5. The table shows the peak positions in the Ca L_II-II_ edge of the NEXAFS spectra shown in (**d**).

**Figure 4:**
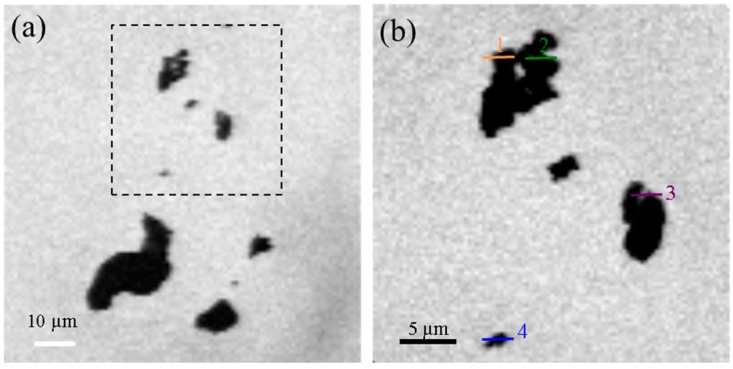

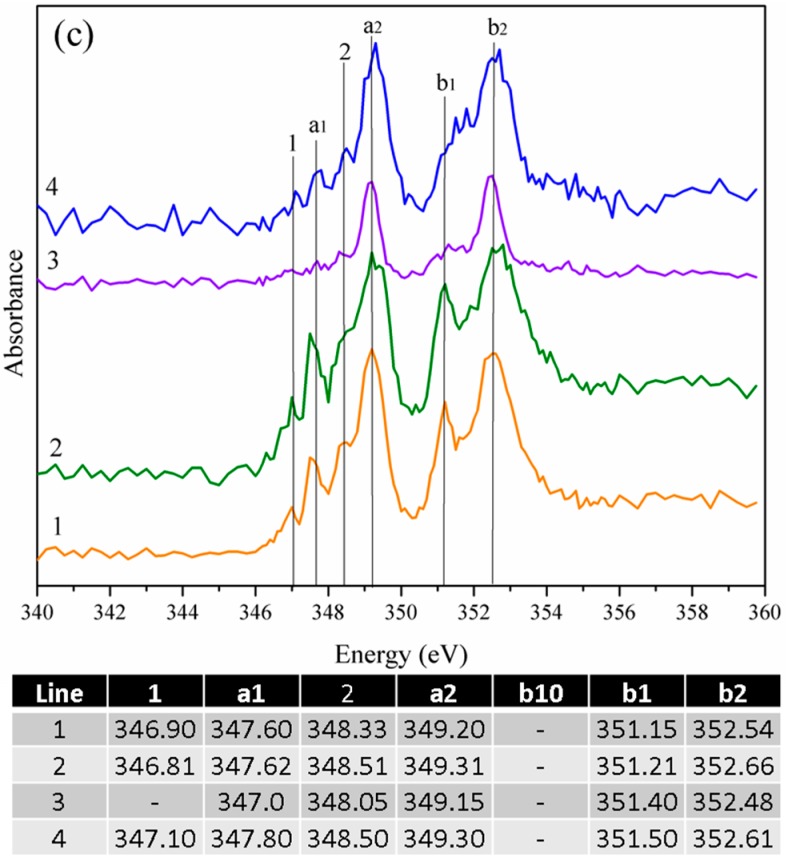
(**a**) Image taken at the calcium L_II,III_ edge of the orthorhombic C_3_A sample; (**b**) calcium L_II,III_ edge of the area underlined in (**a**); and (**c**) NEXAFS spectra of the line scans indicated in (**b**).

The STXM images and their corresponding NEXAFS spectra for the un-reacted cubic and orthorhombic C_3_A samples are shown in Figure 3 and Figure 4, respectively. Ca L_II,III_-edge NEXAFS spectra typically present two major peaks (named a2 and b2), and two minor peaks (named a1 and b1), as well as a few leading peaks (possibly 1, 2) [26]. The STXM images obtained for cubic C_3_A (Figure 3a) show relatively large cubic C_3_A particles agglomerated (>5 μm) primarily with a heterogeneous shape. The NEXAFS spectra at the Ca L_II,III_-edge were undertaken in line-scan mode on the *x*-*y* plane within the lines shown in the areas of greater magnification (Figure 3b,c). The spectra obtained elucidate different peaks as a result of the combined effects of the spin-orbit coupling and crystal field splitting. The larger splitting into two doublets (represented by the highest signals at ~349.2 eV and ~352.5 eV for L_III_ and L_II_, respectively, which are indicated as a_2_ and b_2_, respectively) is the result of spin-orbit coupling. Most of the differences in peak position are slightly less than 0.1 eV, as shown in the table included in Figure 3. However, the peaks in spectra 5 are broader, which indicates a greater thickness around this region. In soft X-ray absorption spectroscopy, this is the main restriction when the spectrum is recorded using transmission mode detection. A small peak at position b_10_ is not observed, but its presence with low intensity cannot be discarded. A detailed description of the peaks can be found elsewhere [26].

Figure 4 shows the STXM images and their corresponding NEXAFS spectra for orthorhombic C_3_A. Similar to cubic-C_3_A, the orthorhombic C_3_A samples exhibited a heterogeneous morphology based on the agglomeration of micro-particles. Their corresponding NEXAFS spectra (Figure 4b) show very clear, small peaks at lower energies than the main peaks (peaks referred to as 1 and 2) and significant differences in the energy of the peak a_1_ in scans taken from different locations in the sample. At the same time, peak a_1_ is located at a slightly lower position compared with the cubic C_3_A samples. The appearance of these multiple peaks is due to the crystal field arising from the symmetry of the atoms surrounding the Ca^2+^ ion in the first coordination sphere [44]. The presence of the main Ca L_III_ and Ca L_II_ peaks with similar intensity shows a well-developed crystalline Ca phase structure [45].

Orthorhombic samples show small and less defined signals at 348.2 and 351.4 eV, which indicate the presence of disordered Ca compounds suggesting that the partial substitution of Na^+^ within the structure can slightly modify its degree of crystallinity. The Ca-L edge spectra reveled slight differences between the micro-scale spatial distributions of Ca compounds for both C_3_A samples, which was primarily observed for those particles with sizes smaller than 2 μm. This result can be attributed to the presence of six types of calcium ions that can be irregularly coordinated with different bond distances [3]. These results are also aligned with the data reported by Geng et al. [26] where Ca atoms within the cubic and orthorhombic C_3_A structure exhibits similar chemical environments, and a slightly greater distortion was identified in the orthorhombic polymorphs. Aluminum in cubic and orthorhombic C_3_A is predominately present in tetrahedral coordination (Al^IV^) sites conforming an arrangement of six-membered rings. Ca occupies the holes between the rings, and six different Ca sites can be identified: three- in six-fold coordination and one of each in seven-, eight-, and nine-fold coordination [3]. The inclusion of Na and its partial substitution for Ca^2+^ cations might also affect the Al-O bond distances, which will exhibit broader ranges than cubic C_3_A samples; however, here, the Al NEXAFS data were not assessed. The distortion of the Ca sites can be associated with the slightly higher Ca L_II_ peak position for the orthorhombic C_3_A (~0.2 eV for peak a_1_, ~0.1 eV for peak a_2_ with an additional slight increase in the b_1_ and b_2_ peaks).

**Figure 5:**
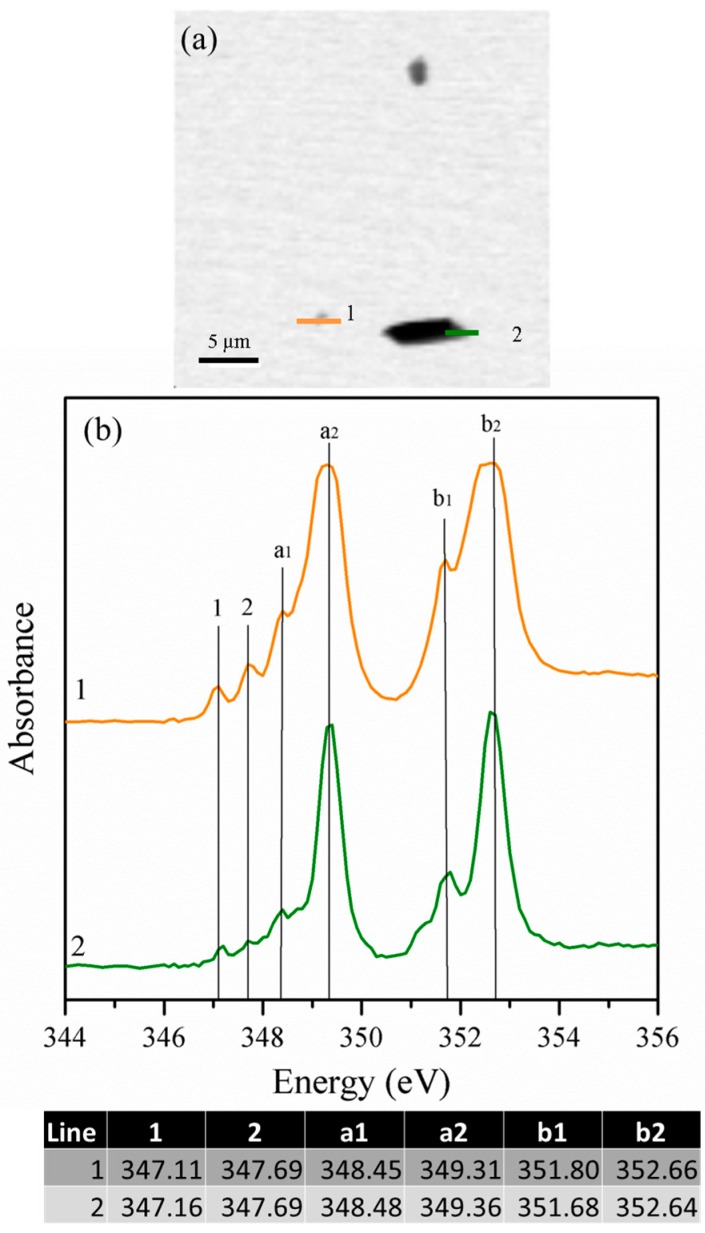
(**a**) Image taken at the calcium L_II,III_ edge of the gypsum sample; and (**b**) NEXAFS of the corresponding line scans.

Gypsum particles exhibited granular crystal shapes (Figure 5a) with sizes even larger than 10 μm. The NEXAFS spectra of gypsum show uniform intensity depending on the region of the particle. Thicker regions may present signs of saturation, which occurs at the Ca edge when the sample is thicker than ~300–500 nm. The BE for all peaks are similar at approximately 349.3 eV and 352.6 eV for Ca L_III_ and L_II_, respectively. As shown in Figure 5c, the NEXAFS spectra of gypsum were collected on a thin (line scan 3) and a thick particle (line scan 2). The ratios of the minor peak intensities to the major peak intensities are greater in spectrum 2 than in spectrum 1 indicating absorption saturation. The peak positions in both spectra are the same. This is consistent with previous reports that the saturation does not change peak positions [20,26]. The spectra also present two small peaks at lower energies (peaks 1 and 2). A minor peak (named b10) appears to be present from spectra 2 (in agreement with the observations from Geng et al. [26]); however, in such a small intensity, the peak fitting cannot recognize it.

### 3.2. Hydration Products of C_3_A in the Presence of Gypsum

#### 3.2.1. Gypsum/Cubic C_3_A Blends with Ratios of 0.20 and 1.19

**Figure 6:**
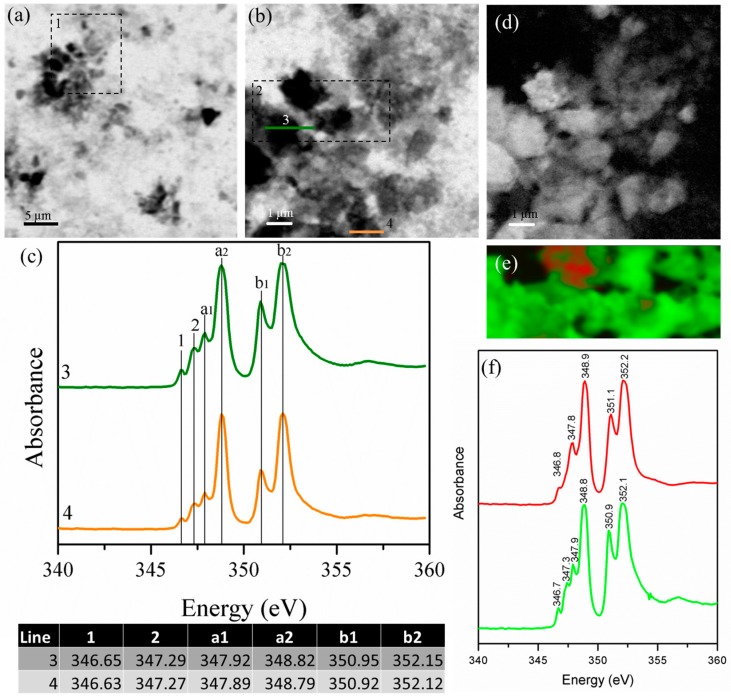
(**a**) Image taken at the calcium L_II,III_ edge of the gypsum/cubic-C_3_A blends at a ratio of 0.20 after three days of curing; (**b**) magnification of area 1 in image (**a**); (**c**) NEXAFS spectra of the line scans indicated in (**b**); (**d**) image maps of calcium in the same area as (**b**), where the brighter areas are related to higher concentrations of calcium; (**e**) Red-green-blue (RGB) composite map of region 2 in image (**b**) (red area is related to ettringite and the green area corresponds to the monosulfate phase); and (**f**) NEXAFS spectra of the red and green regions observed in image (**e**).

Figure 6 shows the STXM images and NEXAFS spectra of the gypsum/cubic-C_3_A blends at a ratio of 0.20 after three days of curing. The STXM images of the sample show that the needle-shaped particles cannot be identified, likely due to the reduced amount of available gypsum. Agreeing with previous reports, when ettringite formed from cubic C_3_A, the samples exhibited a lower aspect ratio than orthorhombic C_3_A [10], meaning smaller and more elongated crystals, even though the gypsum content was different, and the sample was in solution in that case. The Ca L_II-III_ edge spectrum obtained from line scans of the hydrated products (Figure 6b) shown in Figure 6c reveal six different absorption peaks located at approximately 346.6 and 347.3 (peaks 1 and 2) and major peaks located at approximately 347.9 eV, 348.8 eV, 350.9 eV, and 352.15 eV without significant differences in the spatial distribution of the Ca compounds. These results suggest that, at this stage of hydration, monosulfoaluminate is formed and small amounts of ettringite can also be generatedIf there is not enough sulfate available in the solution to achieve an ettringite saturation state, monosulfoaluminate will be formed before even for early age. Based on this, Minard et al. [46] describe the possibility of the formation of both ettringite and AFm-type phases at the very beginning of the hydration process in the presence of gypsum, whereas Merlini et al. [47] describe the evolution of ettringite crystal growth over time through the changes in the lattice parameters. These modifications were identified using a different SO_3_/H_2_O ratio at early stages of hydration when compared with the stoichiometric pure ettringite. Conversely, Meredith et al. [48] observed a gel covering the C_3_A particles in the presence or absence of sulfates. When sulfate is present, the primary monosulfoaluminate will only form in areas of low sulfate content (due to heterogeneities), and ettringite, which has a different morphology, will be formed within the gel. Therefore, the morphology of ettringite crystals can be affected by the water/solid (W/S) ratio where the length and size of the needle-like structure increase with the W/S ratio; however, this possibility is not explored here. Ettringite eventually converts to monosulfoaluminate as the sulfate is fully consumed.

The element map shows the absorption difference below and above the absorption edge and is a direct index of the element concentration. On the element map, a brighter area has a greater concentration of the assessed element (Figure 6d) [30]. A stack leads to a complete scanned area, pixel by pixel, for a determined range of energies (Figure 6e). Therefore, each pixel has spectral information of the composition of the sample at that point. The images are converted to an optical density (OD = ln(*I*_0_/*I*)) using an empty zone in the silicon nitride window of the sample, such as the *I*_0_ region. The Ca L-edge NEXAFS spectra were extracted and used to obtain a RGB (red-green-blue) composite image (shown in Figure 6e) around the square region indicated as 2 in Figure 6b. In this case, only two different calcium species were observed, and for this reason, the third component of the RGB image does not appear. The spatial distribution of Ca-L reveals two different zones whose corresponding NEXFAS spectra show two different signals for Ca-L_II_ with fine absorption structures at 352.2 eV and 352.1 eV (Figure 6f). This elucidates clear differences in the calcium concentration and chemical bonding in different areas. The red areas are related to ettringite, whereas the green area leads to the monosulfate phase, which was also observed by Geng et al. [26]. As there is not enough sulfate in the system, it is possible that monosulfoaluminate is formed after three days of hydration because there is the presence of water for this formation. The a_2_–a_1_ and b_2_–b_1_ distances are very similar to the ones observed by Geng et al. [26] for the pure AFm-type phase’s characterization reinforcing that this is the structure observed here.

According to Kirchheim et al. [10], all C_3_A particles in a saturated lime and gypsum solution hydrate after only a few minutes, nucleating and growing initially inside the grain boundaries. However, later, these particles expand beyond these boundaries, which is the case at this stage. However, their experiments were performed in solution (W/S = 10).

**Figure 7:**
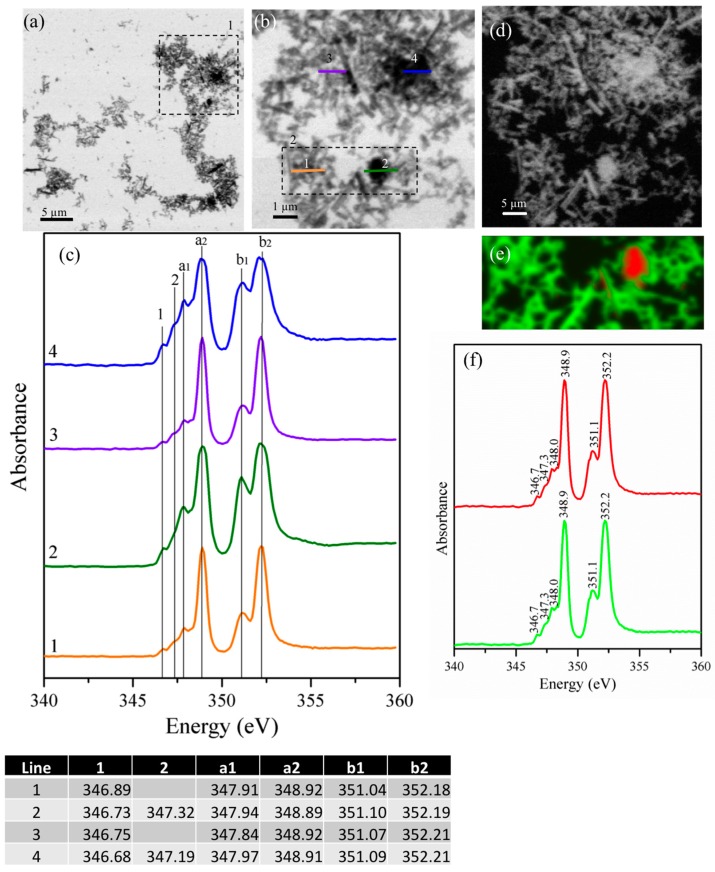
(**a**) Image taken at the calcium L_II,III_ edge of the mix of the cubic C_3_A and the gypsum sample at a ratio of 1.9; (**b**) magnification of area 1 in (**a**); (**c**) NEXAFS spectra of the line scans indicated in (**b**); (**d**) image maps of calcium in the same area as in Figure 6b, where the brighter areas are related to higher concentrations of calcium; (**e**) image stack (red and green areas correspond to ettringite crystals); (**f**) RGB composite map of region 2 in Figure 7b; and (**g**) NEXAFS spectra of the red and green regions observed in (**e**).

**Figure A1:**
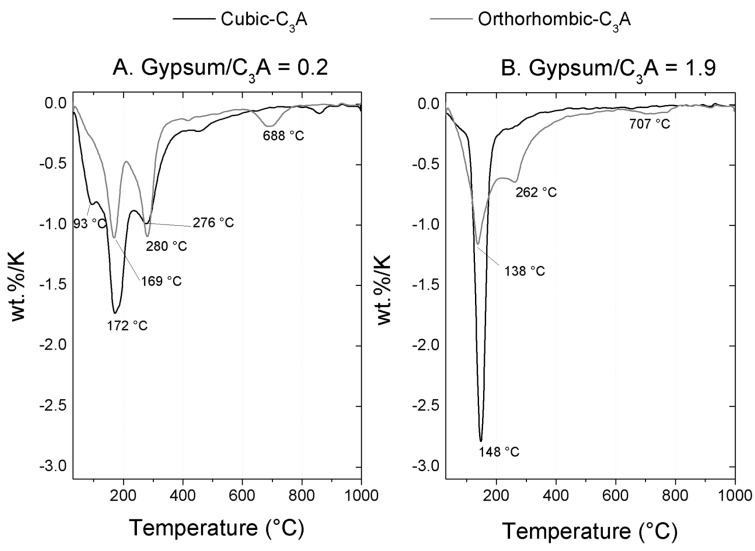
Derivative Thermogravimetric analyses of cubic and orthorhombic C_3_A and gypsum at a ratio of: (**A**) 0.2; and (**B**) 1.9 after three days of curing.

As the content of gypsum is increased (gypsum/cubic-C_3_A blend at a ratio of 1.19), the hydrated products show a more defined needle shape with a greater aspect ratio (Figure 7a,b,d). Images show a fibrous formation distributed densely and are mostly ettringite crystals, which are also clear in the TGA analysis (Appendix A, Figure A1). Some of the more granular particles could be attributed to calcium carbonate, which can be hemicarboaluminate, formed due to the sample carbonation by exposure to air. Additionally, there is still unreacted gypsum in the system, because the reactions happen at a slow rate. According to Kirchheim et al. [15] there is still gypsum left to be consumed after 14 days of hydration in samples of cubic C_3_A paste with the same proportion of gypsum.

The RGB analysis shows the presence of two different calcium species with a slight difference of ~0.2 eV in the FWHM in the peak with a lower absorption fine structure; however, this behavior can be attributed to a different sample thickness, which is related to saturation. In cementitious systems, the only evidences to differentiate phases are the peak positions and the overall shape. These characteristics are more repeatedly observed in both pure single phase samples and mixed samples. Only this can be reliable to distinguish phases, and peak width information cannot be taken into account with reliability because it can simply be an illusion of the saturation effect in this case. Here, the phases cannot be easily defined, and from the spectra, both red and green areas are mostly related to ettringite crystals.

It is remarkable that, even though the morphology is completely unalike, the NEXAFS spectra of the two hydrated samples with different gypsum content were identical. There is a significant shift to a lower energy for the hydrated samples compared with the unhydrated samples, which have Ca L_III_ peaks (b_2_) at approximately 352.3–252.7 eV, whereas the former peak is located at 352.1 eV. In this sense, it is known that the Ca in calcium aluminates has higher BE than the Ca in calcium sulfates, which would explain the decrease in the absorption fine structure for this element due to the combination with sulfates available in the solution during hydration and the formation of the expected hydration product: ettringite, monosulfoaluminate, and perhaps C_3_AH_6_ (in the case where there is a lack of gypsum). These are all reactions that happen essentially from the consumption of gypsum and ettringite when there is still C_3_A in the sample. TGA data presented in Appendix A support this.

#### 3.2.2. Gypsum/Na-Doped C_3_A Blend with Ratios of 0.20 and 1.19

The sample of orthorhombic C_3_A/gypsum blend at ratios of 0.2 and three days of curing do not exhibit a needle-like crystal shape, and clusters particles were identified instead. Kirchheim et al. [10] described these products as hexagonal platelets seen in a plan view appearing as transparent hexagons or, in a lateral view, as long needles. Baquerizo et al. [49] presented images of several different monosulfate formed in the AFm cement phases, which can also be hexagonal in shape. These crystals can be either ettringite, which is because there may not be enough sulfates for this, or C_3_AH_6_, which has a cubic crystal structure and granular/cubic shape and may have been formed together with monosulfoaluminate. However, this can also be a controversial result taking into account that these hexagonal products were only identified at the very early stages of the reaction (<49 min), whereas the products identified here were observed even after at three days of curing. The presence of C_2_AH_8_ cannot be discarded, however this phase is known to be metastable and its conversion to C_3_AH_6_ occurs quickly, especially at temperatures higher than 28 °C [9,50]. The in situ characterization of the hydrated products formed [10] demonstrates a difference compared with the cubic C_3_A, where the needle-like structures grow from the initial grain boundaries, whereas orthorhombic C_3_A hydrates grow from swelling and agglomerating. The results reported here, which are very thin particles, demonstrate the already expected presence of a gel-like structure. This type of product has been observed previously by several authors, mostly in the early hydration stages either for tests in solution [10] or in dry mixtures [48,51] regardless of the gypsum content.

**Figure 8:**
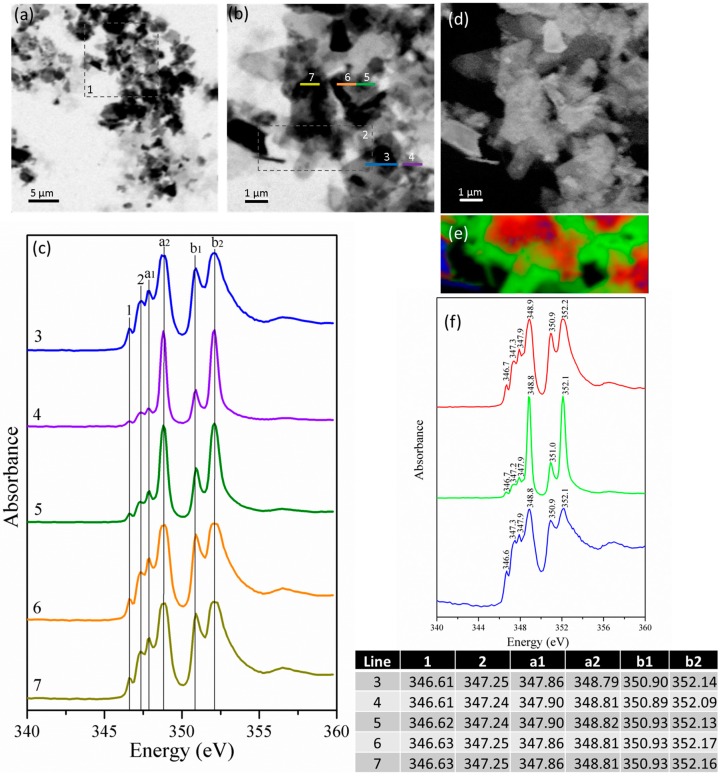
(**a**) Image taken at the calcium L_II,III_ edge of the mix of the orthorhombic C_3_A and gypsum sample at a ratio of 0.2; (**b**) magnification of area 1 in (**a**); (**c**) NEXAFS spectra of the line scans 3–7 indicated in (**b**); (**d**) image maps of calcium in the same area as (**b**), where the brighter areas are related to higher concentration of calcium; (**e**) RGB composite map of region 2 in Figure 8b; and (**f**) NEXAFS spectra of the red, green and blue regions observed in (**e**).

Spectroscopic data for line scans 3 and 4 (Figure 8c) show differences mainly in intensities for areas with different densities presenting some sign of saturation in thicker parts of the particles (e.g., Figure 8c scans 6 and 7). The main peaks of Ca L exhibit slight differences in the absorption of the fine structure (~0.1 eV) among the distinct line scans. Again, image mapping at the calcium edge shows bright regions with high calcium content (Figure 8d). Finally, the RGB image reconstruction (Figure 8e) of the stack region underlined as 2 in Figure 8b shows the presence of three different types of calcium, which is chemically combined in three different ways and distributed in the sample. The spectra show that red is related to the thick part of the sample, which is where the sample is denser. The green area shows very sharp and well-defined calcium peaks, whereas the blue area is smaller and presents broader peaks. However, because green and red spectra are very similar, they are likely both related to monosulfate with different thicknesses. A difference of ~0.1 eV was observed in the Ca L_II_ peaks from different regions, and even though calcium from calcium aluminates have greater absorption fine structure than calcium combined with sulfates, the difference is very slight, and all the three phases appear to be monosulfate.

The peak positions show that there are no remaining unhydrated C_3_A or gypsum, and therefore, all peaks are related to the hydrated phases [26]. The distance between peaks a_2_-a_1_ (approximately 0.95) and b_2_-b_1_ (approximately 1.2) indicates the formation of monosulfate and ettringite with the possible presence of hydrogarnet. These results are in accordance to the observations on TGA analysis (Appendix A, Figure A1).

In orthorhombic C_3_A pastes, ettringite crystals are not observed; however, other products are formed. It is known that metastable phases (C_2_AH_8_ and C_4_AH_19_) are formed after a few minutes of hydration preceding C_3_AH_6_ formation, which, at this point, is fully formed [8,9].

**Figure 9:**
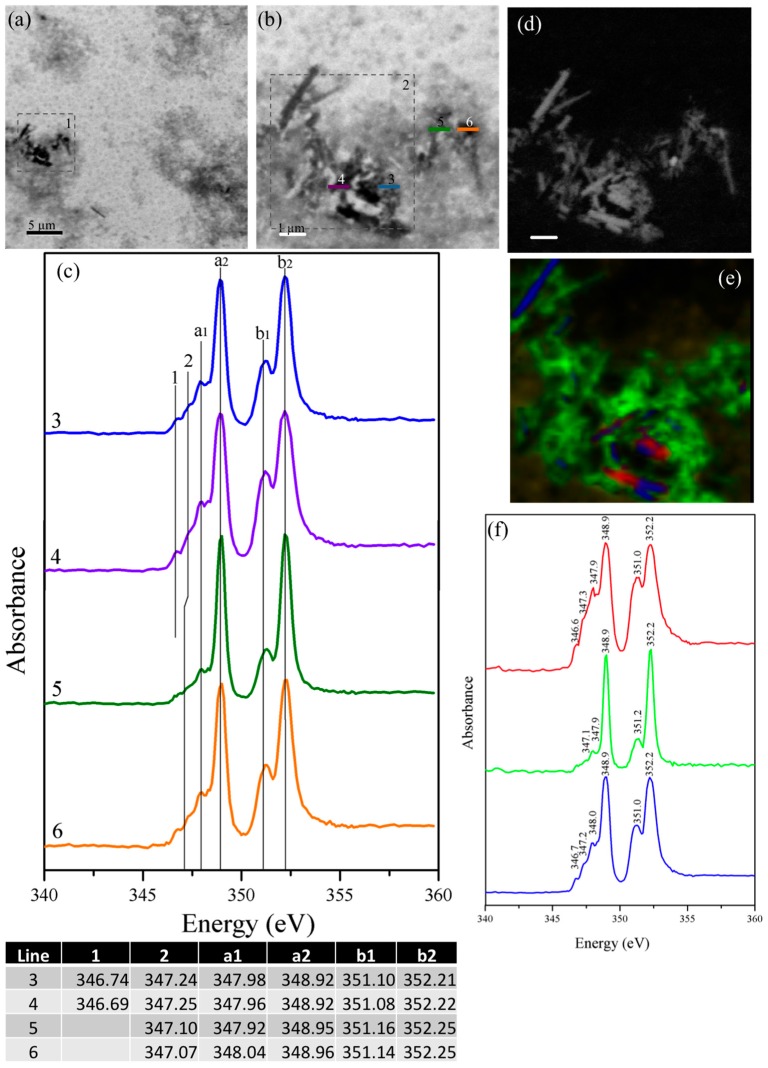
(**a**) Image taken at the calcium L_II,III_ edge of the mix of the orthorhombic C_3_A and gypsum sample at a ratio of 1.9; (**b**) magnification of area 1 in (**a**); (**c**) NEXAFS spectra of line scans 3 and 4 indicated in (**b**); (**d**) image maps of calcium in the same area as (**b**), where the brighter areas are related to higher concentration of calcium; (**e**) RGB composite map of region 2 in Figure 9b; and (**f**) NEXAFS spectra of the red, green and blue regions observed in (**e**).

Figure 9a shows a sample of orthorhombic C_3_A/gypsum with a mass ratio of 1.9 after three days of curing where the particles are agglomerated. Figure 9b shows a greater magnification of the previous image (Figure 9a) whose corresponding line scans are plotted in Figure 9c. Line scans 3 and 4 indicate differences in the particle in 3 compared with the particle in 4, which shows two different regions with no distinctions in the chemical bonding of calcium, as seen in the spectra. Whereas the absorption fine structure is the same for all regions analyzed, the clear differences in the intensity of the peak at the lower absorption fine structure lead to dissimilarities.

The calcium map (Figure 9d) around this region underlines the presence of an agglomerate of needles with different calcium contents, which is clearly seen in the brighter elongated areas. An image stack around region 2 of Figure 9b incurs the presence of three different components in the image (Figure 9f) in terms of the needle shape. The differences in the chemical state of calcium are minimal between the green and red areas, and discrepancies are only evident in the peak shape and intensity of the blue areas with slight differences in the peak energy.

The gel-like structure observed in the sample with a gypsum ratio of 0.2 is not as clear here, and its presence can only be related to the brighter grey areas. The quantity of acicular particles observed for the orthorhombic C_3_A appears to be less than in the cubic samples. In this sample, C_3_A was assumed to have already been fully consumed, while ettringite is observed (and also indicated in the TGA results, Appendix A Figure A1), and some small amounts of unreacted gypsum could be present, as the position of the peak 2 is about 1.1 eV lower than for the unreacted orthorhombic C_3_A (around 348.33 eV versus 347.2 eV for the former) and similar to the peak 2 position in pure gypsum (around 347.7 eV). In the same manner as observed for the cubic C_3_A, the unhydrated orthorhombic has a greater absorption peak energy (e.g., 352.5–352.7 eV for peak b_2_ and the same pattern is observed in the other peaks) than the samples reacted with water and gypsum (352.1–352.3 eV); whereas the morphology for different gypsum contents are alike, the peak positions are very similar indicating the formation of ettringite in all spectra.

## 4. Final Remarks

It is widely known calcium sulfate is added to clinker during cement manufacturing in order to slow down the highly exothermic hydration of C_3_A. The slowed hydration is not fully understood and it is still a reason of debate; different mechanisms are proposed to explain the slow down of the hydration in presence of calcium sulfate. Na_2_O is often incorporated by C_3_A, causing changes in the crystal lattice through the substitution of Ca^2+^ ions by Na^+^ and leading to the formation of the orthorhombic-type structure, and still some work is needed to consistently predict the scope of Na-doped aluminate hydration. In order to achieve a better understand of how the presence of gypsum affect the hydration of cubic and orthorhombic C_3_A, this paper presented an assessment of the chemical and morphological aspects of of synthetic cubic and orthorhombic C_3_A when hydrated at the presence of different contents of gypsum.

The use of synchrotron radiation scanning transmission X-ray microscopy (STXM) along with NEXAFS with a high spatial resolution proved very useful in the experimental study of cements because it provides simultaneous chemical and local micro/nano morphological information in different areas of the sample. This research has provided a significant understanding of the hydration products of cubic and orthorhombic C_3_A with gypsum both chemically and morphologically. Slight differences in the bonding environment of the main elements present in polymorphs C_3_A have been identified using the results obtained by STXM; however, these observations can also be related to structural changes. The importance of having thin particles to permit the X-rays to pass through the sample and avoid spectra saturation became clear and assured reliable results.

For cubic C_3_A, in addition to similarities observed for the samples submitted to the different gypsum content during hydration and with respect to the NEXAFS spectra of the products, the morphology was significantly different for the crystals formed. Additionally, when compared to the anhydrous samples, a strong change in the Ca L_II_ peak positions was observed indicating the transformation from calcium aluminates to calcium sulfoaluminates.

For orthorhombic C_3_A, in the same way, the L_II_ edge of the anhydrous phase was observed to shift to a greater energy than the hydrated samples. Again, differences in morphology for different gypsums available are observed, whereas the NEXAFS spectra were alike.

This work sheds additional light on the complex hydration of different polymorphs of C_3_A, and for a complete understanding of the reactions and products, the STXM data have to be coupled with other techniques.

## Figures and Tables

**Table 1:** Details of the spectral lines recorded by XPS (X-ray Photoelectron Spectroscopy).

Region	Start Energy (eV)	End Energy (eV)	Dwell Time (ms)
**Wide**	0	1200	0.1
**C 1s**	275	295	1
**O 1s**	525	540	0.5
**Ca 2p**	339	359	1
**Al 2p**	65	85	1
**Al 2s**	108	128	1
**Na 1s**	1065	1085	1

**Table 2:** Absorption fine structure and peak width (FWHM) for anhydrous cubic and orthorhombic C_3_A examined by XPS (X-ray Photoelectron Spectroscopy, eV).

Name	Cubic C_3_A		Orthorhombic C_3_A	
	Position	FWHM	Position	FWHM
C 1s	289.4	2.49	289.1	2.14
O 1s	528.6 531.4	1.31 2.50	531.2 532.8 535.7	2.16 2.35 2.43
Ca 2p_3/2_	347.0	2.26	346.7	2.06
Ca 2p_1/2_	350.5	2.20	350.2	2.01
Al 2p	74.1	2.69	74.3	2.76
Na 1s	-	-	1071.7	2.34

## Appendix A

### TGA Analysis

The hydrated C_3_A/gypsum pastes (cubic and orthorhombic) where assessed through termogravimetric analyses (TGA). The hydrated samples after three days of curing were frozen at −196 °C with liquid nitrogen followed by a freezing treatment at −27 °C during 24 h and subsequently lyophilized for 16 h. The TGA was performed using a thermobalance STA409PG from NETZSCH at a heating rate of 10 °C/min up to 1000 °C in a nitrogen atmosphere (60 mL/min).

Based on the TGA results (Figure A1) cubic C_3_A and gypsum at a ratio of 0.2 reported a total weight loss of 33.13%, which is considerably higher when compared to the corresponding system based on orthorhombic C_3_A (20.88%). This is in accordance with the higher reactivity exhibited by the cubic C_3_A in the absence or low content of gypsum [10,11,15,16,17]. Ettringite is identified through the peak around 140 °C, attributed to the loss of water, along with a shoulder at ~250 °C, related to the hydroxylation of aluminum hydroxide [52]. The peaks at 94 °C, 172 °C and 277 °C for the hydrated paste with gypsum/cubic-C_3_A ratio of 0.2 (Figure A1A) is attributed exclusively to the extensive formation of monosulfate-type phases [52]. As expected, higher intensity of the main peak between 130 and 150 °C, attributed mainly to ettringite, is reported by the Cubic-C_3_A systems with higher content of gypsum (Figure A1B). The main peak attributed to ettringite for the sample with gypsum/cubic-C_3_A ratio of 0.20 exhibited a lower intensity, which agrees with the differences of the spatial distributions of Ca-based products identified in the NEXAFS spectra (Figure 6c).

On the other hand, orthorhombic-C_3_A pastes show the presence of two peaks, whose behavior is strongly affected by the gypsum/C_3_A ratio. At lower gypsum content, two peaks with similar intensity are identified at 169 °C and 280 °C, and are attributed to AFm-type phases. Although the presence of some aluminate hydrates (such as C_3_AH_6_) was identified through STXM (Figure 8), DTG curves (Figure A1A) do not show any peak related to its thermal decomposition due to its lower content, when compared to AFm-type phases. Orthorhombic C_3_A presents a peak at 690–700 °C, which could refer to mono/hemi-carboaluminates. This is in accordance to Dubina et al. [38] and happens because orthorhombic C_3_A is more prone to carbonation than the cubic C_3_A.
